# Semi-Supervised Feature Transformation for Tissue Image Classification

**DOI:** 10.1371/journal.pone.0166413

**Published:** 2016-12-02

**Authors:** Kenji Watanabe, Takumi Kobayashi, Toshikazu Wada

**Affiliations:** 1 Department of Information Technology and Human Factors, National Institute of Advanced Industrial Science and Technology (AIST), Tsukuba, Ibaraki, Japan; 2 Department of Computer & Communication Science, Wakayama University, Wakayama, Wakayama, Japan; Worcester Polytechnic Institute, UNITED STATES

## Abstract

Various systems have been proposed to support biological image analysis, with the intent of decreasing false annotations and reducing the heavy burden on biologists. These systems generally comprise a feature extraction method and a classification method. Task-oriented methods for feature extraction leverage characteristic images for each problem, and they are very effective at improving the classification accuracy. However, it is difficult to utilize such feature extraction methods for versatile task in practice, because few biologists specialize in Computer Vision and/or Pattern Recognition to design the task-oriented methods. Thus, in order to improve the usability of these supporting systems, it will be useful to develop a method that can automatically transform the image features of general propose into the effective form toward the task of their interest. In this paper, we propose a semi-supervised feature transformation method, which is formulated as a natural coupling of principal component analysis (PCA) and linear discriminant analysis (LDA) in the framework of graph-embedding. Compared with other feature transformation methods, our method showed favorable classification performance in biological image analysis.

## Introduction

In biological image analysis, biologists manually identify and/or classify the images captured via a microscope. However, the data usually comprise a large number of images, and thus the analysis imposes a heavy burden on biologists, which increases the risk of false annotations. Therefore, in order to improve both efficiency and accuracy, there is a great demand for developing a system to support biologists with image annotation.

Recently, many such systems have been proposed [1–5], and some of them are currently being used in biological and medical research. These supporting systems, which analyze biological images, are generally constructed based on feature extraction and classification methods. In those systems, task-oriented feature extraction methods, such as by using the shift-and-rotation-invariant feature extraction method for classifying biological particles [4], are very effective [1–4] at improving the classification accuracy. However, the improvement is limited when the method is applied to an unexpected task (such as when a feature extraction method for intracellular particles is applied to an image classification task for tissues) [6], and knowledge of Computer Vision and/or Pattern Recognition is necessary in order to successfully apply the various feature extraction methods. Unfortunately, few of the primary users of these systems, the research biologists, specialize in Computer Vision and/or Pattern Recognition.

In recent years, the methods of deep learning such as convolutional neural networks (CNN) have produced promising performance in many image classification tasks [7, 8]. For training those CNN-based methods, it is necessary to prepare large-scale datasets as well as specialized knowledge about the CNN architectures, which however is generally not available in the field of biological classification. On the other hand, the CNN feature extractors “pre-trained” on the large-scale data, e.g., ImageNet [9], of different domain are shown to be transferable by effectively improving, e.g., medical image classification [10]. In that case, it will be further useful to apply a (semi-) supervised feature transformation method that can automatically adapt the general features to various types of tasks by making these methods available to biologists lacking in specialized knowledge of feature extraction methods.

Here, we simply define that the feature transformation as the linear mapping of ***y*** = *A*^T^***x***, in which the transformation matrix *A* is obtained by solving an optimization problem. We can apply the above feature transformation to obtain classifiable features ***y*** from various characteristics features ***x*** by using *A* without knowing how ***x*** is constructed. Therefore, we can regard a multivariate analysis as the feature transformation.

When we apply the feature transformation to the extracted features in the classification of biological datasets, the feature transformation method should be applicable to the ill-posed problem without the specialized knowledge, because the biological dataset is generally small compared to the dimensionality of the input vector as shown in [11]. In this case, the multivariate analysis method can easily deal with the ill-posed problem by solving a dual formulation.

Principal component analysis (PCA) uses a simple unsupervised feature transformation, and it is widely used for applications requiring dimensionality reduction and/or feature extraction [12]. It is essentially the same as the Karhunen-Loève transformation [13], and it is formulated as the problem of estimating the orthogonal transformation coefficients from a given set of input data by maximizing the variance of the transformed data. Some studies have shown that when the size of the training dataset is small, PCA can outperform LDA, and in addition, PCA is less sensitive to differences in the categories [14]. However, in general, (semi-) supervised feature transformations perform better than PCA.

Fishers linear discriminant analysis (LDA) [15] is a well-known method for extracting the features that maximize the discrimination. LDA is formulated as the problem of estimating the transformation coefficients for labeled input data such that the ratio of the between-class variance to the within-class variance is maximized. When the label information is available, e.g., in classification tasks, LDA performs better than PCA [16]. However, especially in the biological field, it is difficult to prepare many training samples which were given reliable class labels. When the number of labeled samples is less than the number of dimensions, the covariance matrix of the classes may not be accurately estimated. In this case, the generalization performance for the testing samples cannot be guaranteed. In order to overcome this problem, various feature transformation methods have been proposed; these include semi-supervised discriminant analysis (SDA) [17] and the heuristic fusion algorithm [18].

For biological data such as tissue images, the given class labels are often unreliable, because objects to be measured inherently contain some physical and biological uncertainty. Moreover, some given labels might be incorrectly assigned by human intuition. Whereas, reliable labels would be available for a small portion of the training samples. In such case, the method of semi-supervised learning is effectively applied to transform the features extracted from the biological data and/or it.

SDA is a natural extension of LDA in a graph-embedding framework [19]. The graph-embedding framework can be considered as a general expression of multivariate analysis, such as PCA and LDA, in a graph structure. The regularization term in SDA is based on the locality preserving projections (LPP) [20, 21] and is introduced to deal with the unlabeled training samples. Thus, it efficiently exploits both labeled and unlabeled data; the labeled data are used to maximize the discriminating power, while the unlabeled data are used to maximize the locality preserving power. When applied to actual data, especially when applied to biological microscopic images, it is difficult to determine the optimal similarity measure for the regularization term, because this depends on the characteristics of the sample.

In this paper, we propose semi-supervised component analysis (SCA), a method for transforming features in order to improve the classification accuracy and the usability of image analysis in biological fields. Our method is formulated in the framework of semi-supervised learning, directly incorporating PCA and LDA via a graph-embedding expression; a discriminant criterion is added to the PCA when there are labeled training samples. This is not the same as the fusion algorithm [18], which heuristically and individually mixes the coefficients estimated by LDA and PCA, and this ensures that our proposed method performs at least as well as either PCA or LDA. In addition, our method does not require a priori knowledge of similarity, as does SDA. Furthermore, we also present a kernel-based method (similar to those used in [19–21]) to deal with ill-posed problems.

A preliminary version of the proposed SCA has been published [22]. In the present paper, we propose a refined version and discuss its formulation. In addition, we introduce a scaling parameter to the definition of the SCA in order to improve the cooperation between PCA and LDA.

## Methods

In this section, we briefly review PCA and LDA expressed by the graph-embedding framework, and we then present SCA.

### Principal component analysis

PCA is a linear transformation method that is widely used to estimate the orthogonal bases so as to maximize the variance of projected data. Suppose *X* = [***x***_1_ … ***x***_*n*_] ∈ ℜ^*m*×*n*^ be an input dataset, where ***x***_*i*_ is an *m*-dimensional vector for the *i*-th feature. PCA constructs a linear mapping *A* ∈ ℜ^*m*×*r*^ from the input vector ***x*** to a new feature vector ***y*** of lower dimensionality (*r* < *m*), as follows:

$$y=A^{\text{T}}x.$$ (1)

The optimal transformation matrix A* is obtained by maximizing the following objective function:

$$\begin{array}{l} A^{*}=\text{arg}\underset{A}{\text{max }}tr\left(A^{\text{T}}\Sigma A\right)\text{ , }\\ \text{s.t. }A^{\text{T}}A=I\text{ ,} \end{array}$$ (2)

where

$$\Sigma =\frac{1}{n}\sum_{i}^{n}\left(x_{i}-\mu \right){\left(x_{i}-\mu \right)}^{\text{T}},$$ (3)

*n* is the number of samples, and ***μ*** is the sample mean vector. In the graph-embedding framework, the covariance matrix Σ can be reformulated as follows [19]:

$$\begin{array}{l} \Sigma =\frac{1}{n}X\left(I-\frac{1}{n}ee^{\text{T}}\right)X^{\text{T}}\\ \;=XL_{t}X^{\text{T}}, \end{array}$$ (4)

where ***e*** = [1 … 1]^T^ is an *n*-dimensional vector, and *L*_*t*_ is the Laplacian matrix for the total covariance. From Eqs (2) and (4), A* can be obtained by solving the following eigenvalue problem:

$$XL_{t}X^{\text{T}}A=A\Lambda \text{ ,}$$ (5)

where Λ = diag(*λ*_1_,…, *λ*_*r*_) is a diagonal matrix of eigenvalues.

### Linear discriminant analysis

Fisher [15] proposed LDA, which determines the subspace that maximizes the ratio of the between-class variance to the within-class variance.

Let *X* be a training dataset, such that ***x*** belongs to one of the *k* classes {*c*_1_, …, *c*_*k*_}, and suppose that each member of *X* is labeled according to the class to which it belongs. Then, LDA constructs the linear transformation given in Eq (1).

Based on the discriminant criterion, the optimal transformation matrix A* is obtained by maximizing the following objective function:

$$\begin{array}{l} A^{*}=\text{arg}\underset{A}{\text{max }}tr\left({\left(A^{\text{T}}\Sigma_{w}A\right)}^{-1}A^{\text{T}}\Sigma_{b}A\right)\text{ , }\\ \text{s.t. }A^{\text{T}}\Sigma_{w}A=I, \end{array}$$ (6)

where

$$\Sigma_{w}=\frac{1}{n}\sum_{q}^{k}\sum_{i}^{n_{q}}\left(x_{qi}-\mu_{q}\right){\left(x_{qi}-\mu_{q}\right)}^{\text{T}},$$ (7)

$$\Sigma_{b}=\sum_{q}^{k}\frac{n_{q}}{n}\left(\mu_{q}-\mu \right){\left(\mu_{q}-\mu \right)}^{\text{T}},$$ (8)

*n*_*q*_ is the number of samples in the *q*-th class, ***x***_*qi*_ is the *i*-th input vector in the *q*-th class, and ***μ***_*q*_ is the mean vector of the *q*-th class. In the graph-embedding framework, the within-class covariance matrix Σ_*w*_ and the between-class covariance matrix Σ_*b*_ can be reformulated as follows [19]:

$$\begin{array}{l} \Sigma_{w}=\frac{1}{n}X\text{diag}\left(I-\frac{1}{n_{1}}e_{1}e_{1}{}^{\text{T}},\ldots ,I-\frac{1}{n_{k}}e_{k}e_{k}{}^{\text{T}}\right)X^{\text{T}}\\ \;=XL_{w}X^{\text{T}}, \end{array}$$ (9)

$$\begin{array}{l} \Sigma_{b}=\Sigma -\Sigma_{w}\\ \;=X\left(L_{t}-L_{w}\right)X^{\text{T}}\\ \;=XL_{b}X^{\text{T}}, \end{array}$$ (10)

where ***e***_*q*_ = [1 … 1]^T^ is an *n*_*q*_-dimensional vector, and *L*_*w*_, and *L*_*b*_ are the Laplacian matrices for the within-class covariance and for the between-class covariance, respectively. From Eqs (6), (9) and (10), A* can be obtained by solving the following generalized eigenvalue problem:

$$XL_{b}X^{\text{T}}A=XL_{w}X^{\text{T}}A\Lambda \text{ .}$$ (11)

### Semi-supervised component analysis

We propose an efficient method for transforming features; it is based on PCA, which directly uses a discriminant criterion for labeled input data. Our method, SCA, can be formulated as a natural coupling of PCA and LDA via a graph-embedding expression; the graph structure is directly determined from the distributions of the labeled and unlabeled samples. The objective function of SCA is essentially expressed by the sum of the Laplacian matrices which are defined in the functions of PCA and LDA. Where, the Laplacian matrices are the graphs of the total variance for unlabeled samples and the between-class covariance for labeled samples as shown in the next section. Those variance and covariance are calculated referring to the mean vector which averages the labeled and unlabeled samples. SCA proposes a semi-supervised feature transformation, and it is not necessary that all training samples have class labels in order to obtain an appropriate feature transformation matrix.

Suppose we have an input dataset *X* = [*X*_*l*_ *X*_*u*_] ∈ ℜ^*m*×*n*^, such that *n* = *n*_*l*_ + *n*_*u*_, where *l* and *u* denote the labeled set and the unlabeled set, respectively. Then, SCA constructs the linear transformation shown in Eq (1), and the optimal transformation matrix A* can be obtained by maximizing the following objective function:

$$A^{*}=\text{arg }\underset{A}{\text{max}}tr\left(A^{\text{T}}XLX^{\text{T}}A\right),$$ (12)

$$\text{s.t. }\alpha \frac{n_{u}}{n}A^{\text{T}}A+\frac{n_{l}}{n}A^{\text{T}}X{\hat{L}}_{w}X^{\text{T}}A=I,$$ (13)

where *α* is a scaling parameter. From Eq (12), A* can be obtained by solving the following generalized eigenvalue problem:

$$XLX^{\text{T}}A=\left\{\alpha \frac{n_{u}}{n}I+\frac{n_{l}}{n}X{\hat{L}}_{w}X^{\text{T}}\right\}A\Lambda .$$ (14)

The Laplacian matrix for the within-class covariance ${\hat{L}}_{w}$ in Eq (12) is calculated from only the labeled input samples, based on Eq (9). The Laplacian matrix *L* in Eq (12) is constructed from Eqs (4) and (9), and it is defined as follows:

$$\begin{array}{l} L=\left[\begin{array}{cc} L_{ll} & L_{lu}\\ L_{lu} & L_{uu} \end{array}\right]\\ \;=L_{t}-\left[\begin{array}{cc} \frac{n_{l}}{n}L_{w} & 0\\ 0 & 0 \end{array}\right]\\ \;=\frac{1}{n}\left[\begin{array}{cc} -\frac{1}{n}e_{l}e_{l}{}^{\text{T}}+\text{diag}\left(\frac{1}{n_{1}}e_{1}e_{1}{}^{\text{T}},\ldots ,\frac{1}{n_{k}}e_{k}e_{k}{}^{\text{T}}\right) & -\frac{1}{n}e_{l}e_{u}{}^{\text{T}}\\ -\frac{1}{n}e_{u}e_{l}{}^{\text{T}} & I-\frac{1}{n}e_{u}e_{u}{}^{\text{T}} \end{array}\right], \end{array}$$ (15)

where ***e***_*l*_ and ***e***_*u*_ are components of ***e***^T^ = [***e***_*l*_^T^ ***e***_*u*_^T^]. From Eq (15), the sample variance matrix *XLX*^T^ and the within-class variance matrix $X{\hat{L}}_{w}X^{Τ}$ in SCA are reformulated as follows:

$$\left\{ \begin{array}{l} XLX^{\text{T}}=\left[\begin{array}{cc} X_{l} & X_{u} \end{array}\right]\left[\begin{array}{cc} L_{ll} & L_{lu}\\ L_{ul} & L_{uu} \end{array}\right]{\left[\begin{array}{cc} X_{l} & X_{u} \end{array}\right]}^{\text{T}}\\ \;=\Sigma -\frac{n_{l}}{n}\Sigma_{w}\\ X{\hat{L}}_{w}X^{\text{T}}=\left[\begin{array}{cc} X_{l} & X_{u} \end{array}\right]\left[\begin{array}{cc} L_{w} & 0\\ 0 & 0 \end{array}\right]{\left[\begin{array}{cc} X_{l} & X_{u} \end{array}\right]}^{\text{T}}\\ \;=X_{l}L_{w}X_{l}{}^{\text{T}}\\ \;=\Sigma_{w} \end{array}\right.,$$ (16)

where $X_{l}L_{w}X_{l}{}^{\text{T}}=\frac{1}{n_{l}}\sum_{q}^{k}\sum_{i\in C_{q}}^{n_{q}}\left(x_{qi}-\mu_{q}\right){\left(x_{qi}-\mu_{q}\right)}^{\text{T}}$ is the within-class covariance matrix for the labeled samples, *C*_*q*_ is the labeled subset in the *q*-th class, and *$n_{l}=\sum_{q}^{k}n_{q}$*.

The difference between the scales of the first and second terms in Eq (13) is likely to cause the feature transformation to be unstable in terms of the classification accuracy. When the within-class covariance matrix Σ_*w*_ is the same as the identity matrix, it is reasonable to set *α* = 1. However, it is impossible to determine whether the within-class covariance matrix is similar to the identity matrix for the raw data.

Here, we rewrite the constraint of SCA in Eq (12) as follows:

$$\alpha \frac{n_{u}}{n}A^{\text{T}}A+\frac{n_{l}}{n}A^{\text{T}}XL_{w}X^{\text{T}}A=\frac{n_{u}}{n}A^{\text{T}}\left(\alpha I-\Sigma_{w}\right)A+A^{\text{T}}\Sigma_{w}A,$$ (17)

and when (*αI* − Σ_*w*_) is approximated as Σ_*b*_, the first term of right-hand equation satisfies the positive definiteness. Then, the weighted identity matrix *αI* can be approximated as following equation:

$$\begin{array}{ll} \alpha I & ≅\Sigma_{b}+\Sigma_{w}\\ & =\Sigma_{t}≅\Sigma \end{array},$$ (18)

where Σ is the total covariance matrix.

Based on the above discussion, we determined the scaling parameter to be *α = tr*(Σ) / *m* in order to achieve a scale-invariant feature transformation.

From the above definitions, it can be seen that SCA is equal to LDA when all of the training samples are labeled (*n*_*l*_ = *n*), and it is equivalent to PCA when all of the training samples are unlabeled (*n*_*u*_ = *n*).

### Discussion of SCA

In this section, we discuss SCA. It is worth pointing out that the covariance matrix *XLX*^T^ in Eq (12) can be represented by a linear combination of a between-class scatter matrix for the labeled samples *S*_*b*_ and a total scatter matrix for the unlabeled samples *S*_*u*_, when the scatter is defined relative to the total mean vector, as follows:

$$\begin{array}{l} S_{b}=\sum_{q}^{k}n_{q}\left(\mu_{q}-\mu \right){\left(\mu_{q}-\mu \right)}^{\text{T}}\\ \;=\sum_{q}^{k}n_{q}\mu_{q}\mu_{q}{}^{\text{T}}-\sum_{q}^{k}n_{q}\mu \mu_{q}{}^{\text{T}}-\sum_{q}^{k}n_{q}\mu_{q}\mu^{\text{T}}+\sum_{q}^{k}n_{q}\mu \mu^{\text{T}}\\ \;=\sum_{q}^{k}\left\{\left(\sum_{i\in C_{q}}^{n_{q}}x_{i}\right)\left(\sum_{j\in C_{q}}^{n_{q}}\frac{1}{n_{q}}x_{j}{}^{\text{T}}\right)\right\}-\left(\sum_{i}^{n}\frac{1}{n}x_{i}\right)\left(\sum_{j\in l}^{n_{{}_{l}}}x_{j}{}^{\text{T}}\right)\\ \;-\left(\sum_{i\in l}^{n_{{}_{l}}}x_{i}\right)\left(\sum_{j}^{n}\frac{1}{n}x_{j}{}^{\text{T}}\right)+n_{l}\left(\sum_{i}^{n}\frac{1}{n}x_{i}\right)\left(\sum_{j}^{n}\frac{1}{n}x_{j}{}^{\text{T}}\right)\\ \;=\left[\begin{array}{cc} X_{l} & X_{u} \end{array}\right]\left[\begin{array}{cc} \text{diag}\left(\frac{1}{n_{1}}e_{1}e_{1}{}^{\text{T}},\ldots ,\frac{1}{n_{k}}e_{k}e_{k}{}^{\text{T}}\right)+\frac{-2n+n_{l}}{n^{2}}e_{l}e_{l}{}^{\text{T}} & \frac{-n_{u}}{n^{2}}e_{l}e_{u}{}^{\text{T}}\\ \frac{-n_{u}}{n^{2}}e_{u}e_{l}{}^{\text{T}} & \frac{n_{l}}{n^{2}}e_{u}e_{u}{}^{\text{T}} \end{array}\right]{\left[\begin{array}{cc} X_{l} & X_{u} \end{array}\right]}^{Τ}, \end{array}$$ (19)

$$\begin{array}{l} S_{u}=\sum_{i\in u}^{n_{u}}\left(x_{i}-\mu \right){\left(x_{i}-\mu \right)}^{\text{T}}\\ \;=\sum_{i\in u}^{n_{u}}x_{i}x_{i}{}^{\text{T}}-\sum_{i\in u}^{n_{u}}x_{i}\mu^{\text{T}}-\sum_{i\in u}^{n_{u}}\mu x_{i}{}^{\text{T}}+\sum_{i\in u}^{n_{u}}\mu \mu^{\text{T}}\\ \;=\sum_{i\in u}^{n_{u}}x_{i}x_{i}{}^{\text{T}}-\left(\sum_{i\in u}^{n_{u}}x_{i}\right){\left(\sum_{j}^{n}\frac{1}{n}x_{j}\right)}^{\text{T}}-\left(\sum_{j}^{n}\frac{1}{n}x_{j}\right){\left(\sum_{i\in u}^{n_{u}}x_{i}\right)}^{\text{T}}+n_{u}\left(\sum_{i}^{n}\frac{1}{n}x_{i}\right){\left(\sum_{j}^{n}\frac{1}{n}x_{j}\right)}^{\text{T}}\\ \;=\left[\begin{array}{cc} X_{l} & X_{u} \end{array}\right]\left[\begin{array}{cc} \frac{n_{u}}{n^{2}}e_{l}e_{l}{}^{\text{T}} & \frac{-n+n_{u}}{n^{2}}e_{l}e_{u}{}^{\text{T}}\\ \frac{-n+n_{u}}{n^{2}}e_{u}e_{l}{}^{\text{T}} & I+\frac{-2n+n_{u}}{n^{2}}e_{u}e_{u}{}^{\text{T}} \end{array}\right]{\left[\begin{array}{cc} X_{l} & X_{u} \end{array}\right]}^{Τ}. \end{array}$$ (20)

From Eqs (15), (19) and (20), the covariance matrix *XLX*^T^ in Eq (12) can be rewritten as follows:

$$\begin{array}{ll} XLX^{Τ} & \frac{1}{n}\left[\begin{array}{cc} X_{l} & X_{u} \end{array}\right]\left[\begin{array}{cc} -\frac{1}{n}e_{l}e_{l}{}^{\text{T}}+\text{diag}\left(\frac{1}{n_{1}}e_{1}e_{1}{}^{\text{T}},\ldots ,\frac{1}{n_{k}}e_{k}e_{k}{}^{\text{T}}\right) & -\frac{1}{n}e_{l}e_{u}{}^{\text{T}}\\ -\frac{1}{n}e_{u}e_{l}{}^{\text{T}} & I-\frac{1}{n}e_{u}e_{u}{}^{\text{T}} \end{array}\right]{\left[\begin{array}{cc} X_{l} & X_{u} \end{array}\right]}^{Τ}\\ & =\frac{1}{n}\left[\begin{array}{cc} X_{l} & X_{u} \end{array}\right]\left[\begin{array}{cc} \text{diag}\left(\frac{1}{n_{1}}e_{1}e_{1}{}^{\text{T}},\ldots ,\frac{1}{n_{k}}e_{k}e_{k}{}^{\text{T}}\right)+\frac{-2n+n_{l}+n_{u}}{n^{2}}e_{l}e_{l}{}^{\text{T}} & \frac{-n_{u}-n+n_{u}}{n^{2}}e_{l}e_{u}{}^{\text{T}}\\ \frac{-n_{u}-n+n_{u}}{n^{2}}e_{u}e_{l}{}^{\text{T}} & I+\frac{n_{l}-2n+n_{u}}{n^{2}}e_{u}e_{u}{}^{\text{T}} \end{array}\right]{\left[\begin{array}{cc} X_{l} & X_{u} \end{array}\right]}^{Τ}\\ & \begin{array}{l} =\frac{1}{n}\left[\begin{array}{cc} X_{l} & X_{u} \end{array}\right]\left[\begin{array}{cc} \text{diag}\left(\frac{1}{n_{1}}e_{1}e_{1}{}^{\text{T}},\ldots ,\frac{1}{n_{k}}e_{k}e_{k}{}^{\text{T}}\right)+\frac{-2n+n_{l}}{n^{2}}e_{l}e_{l}{}^{\text{T}} & \frac{-n_{u}}{n^{2}}e_{l}e_{u}{}^{\text{T}}\\ \frac{-n_{u}}{n^{2}}e_{u}e_{l}{}^{\text{T}} & \frac{n_{l}}{n^{2}}e_{u}e_{u}{}^{\text{T}} \end{array}\right]{\left[\begin{array}{cc} X_{l} & X_{u} \end{array}\right]}^{Τ}\\ \;+\frac{1}{n}\left[\begin{array}{cc} X_{l} & X_{u} \end{array}\right]\left[\begin{array}{cc} \frac{n_{u}}{n^{2}}e_{l}e_{l}{}^{\text{T}} & \frac{-n+n_{u}}{n^{2}}e_{l}e_{u}{}^{\text{T}}\\ \frac{-n+n_{u}}{n^{2}}e_{u}e_{l}{}^{\text{T}} & I+\frac{-2n+n_{u}}{n^{2}}e_{u}e_{u}{}^{\text{T}} \end{array}\right]{\left[\begin{array}{cc} X_{l} & X_{u} \end{array}\right]}^{Τ} \end{array}\\ & =\frac{1}{n}\left(S_{b}+S_{u}\right). \end{array}$$ (21)

From the above analysis, we see that by centering the total sample mean, SCA maximizes the between-class discrimination of the labeled samples and minimizes the information loss of the unlabeled samples.

### Kernel extension for ill-posed problems

Suppose that a feature transformation method is applied to a dataset that is small compared to the dimensionality of the input vector (e.g., text mining or image recognition using raw data). In this case, it is necessary to extend the SCA to include a nonlinear method in order to make the problem feasible, resulting in a dual formulation [19–21].

In SCA, let *Φ*(*X*) denote the input feature matrix in the Hilbert space, where *X* = [*X*_*l*_ *X*_*u*_] and *Φ*(*X*) = [*φ*(***x***_1_) … *φ*(***x***_*n*_)]. The generalized eigenproblem in the Hilbert space can be written as follows:

$$\Phi \left(X\right)L\Phi {\left(X\right)}^{\text{T}}A=\left\{\alpha \frac{n_{u}}{n}I+\frac{n_{l}}{n}\Phi \left(X\right)\left[\begin{array}{cc} L_{w} & 0\\ 0 & 0 \end{array}\right]\Phi {\left(X\right)}^{\text{T}}\right\}A\Lambda .$$ (22)

We formulate the nonlinear case in a way that uses the dot product exclusively. Therefore, we consider the expression of the dot product on the Hilbert space, as given by the following kernel function:

$$K\left(x_{i},\;x_{j}\right)=\varphi {\left(x_{i}\right)}^{Τ}\varphi \left(x_{j}\right),$$ (23)

because the eigenvectors in Eq (22) are a linear combination of ***a*** = *Φ*(*X*)***b***, and *K* = *Φ*(*X*)^T^*Φ*(*X*). By simplifying the notation, we obtain the following generalized eigenvalue problem:

$$KLK^{\text{T}}B=\left\{\alpha \frac{n_{u}}{n}K+\frac{n_{l}}{n}K\left[\begin{array}{cc} L_{w} & 0\\ 0 & 0 \end{array}\right]K^{\text{T}}\right\}B\Lambda .$$ (24)

## Results

### Comparative evaluation of SCA

In order to confirm the effectiveness of our proposed method, SCA, we conducted experiments to compare the relative accuracy by using well-known machine learning repositories [23]. Table 1 shows a summary of the datasets in the repository: *Satimage*, *Shuttle*, *Optdigits*, *Pendigits*, and *Isolet* [23]. The training data and the test data were sampled according to the indicated distributions.

**Table 1:** Benchmark datasets for classification.

	# of class	# of training	# of test	# of attribute
*Satimage*	6	4,435	2,000	36
*Shuttle*	7	43,500	14,500	8
*Optdigits*	10	3,823	1,797	64
*Pendigits*	10	7,494	3,498	16
*Isolet*	26	6,238	1,559	617

The classification accuracies were evaluated on the given train/test splits in each of the datasets. In the training dataset, the ratio of unlabeled samples, denoted by *β*, is changed in {0, 0.01, 0.02, …, 0.99, 1}. The training dataset was randomly split into labeled and unlabeled samples at {*β* | 0 < *β <* 1}, which was repeated ten times. We evaluate the classification performance by using the mean recognition rates and the standard deviations over the ten times trials.

The classification accuracies for test datasets were calculated by applying the instance-based classifier, namely the nearest-neighbor (NN) classifier, with all the labels of training samples in the transformed feature space and using full-rank coefficients in order to fairly compare the feature transformation methods.

We confirmed the performance of SCA in which *α* was set to {*α* | 0.01, 0.1, 1.0, 10.0, *tr*(Σ) / *m*}. Table 2 shows the highest mean recognition rates and the standard deviations at each *α* in the range of 0.01 ≤ *β* ≤ 0.99. For the datasets excluding *Isolet*, the SCA by setting our proposed parameter, *α = tr*(Σ) / *m*, shows the best classification performances. For *Isolet*, the mean recognition rates at all *α* were over 0.92. From the results, our proposed method is useful because it derives better recognition rates without the parameter search for *α*.

**Table 2:** Recognition rates with benchmark dataset at each *α*.

	*Satimage*	*Shuttle*	*Optdigits*	*Pendigits*	*Isolet*
*α* = 0.01	0.7809 (± 0.0146) [*β* = 0.99]	**0.9992** **(± 0.0000)** [*β* = 0.33]	0.9693 (± 0.0015) [*β* = 0.88]	0.9723 (± 0.0001) [*β* = 0.01]	0.9210 (± 0.0059) [*β* = 0.01]
*α* = 0.1	0.8429 (± 0.0091) [*β* = 0.99]	**0.9992** **(± 0.0000)** [*β* = 0.04]	0.9784 (± 0.0019) (*β* = 0.99)	0.9723 (± 0.0001) [*β* = 0.01]	0.9239 (± 0.0053) [*β* = 0.01]
*α* = 1.0	0.8879 (± 0.0055) [*β* = 0.99]	**0.9992** **(± 0.0000)** [*β* = 0.03]	0.9817 (± 0.0015) [*β* = 0.97]	0.9738 (± 0.0007) [*β* = 0.93]	0.9317 (± 0.0024) [*β* = 0.01]
*α* = 10.0	0.8969 (± 0.0023) [*β* = 0.99]	**0.9992** **(± 0.0000)** [*β* = 0.01]	**0.9827** **(± 0.0009)** [*β* = 0.75]	0.9759 (± 0.0006) [*β* = 0.99]	**0.9373** **(± 0.0034)** [*β* = 0.01]
*α = tr*(Σ) / *m*	**0.8985** **(± 0.0008)** [*β* = 0.90]	**0.9992** **(± 0.0000)** [*β* = 0.01]	**0.9827** **(± 0.0007)** (*β* = 0.68)	**0.9774** **(± 0.0001)** [*β* = 0.82]	0.9259 (± 0.0045) [*β* = 0.01]

**Fig 1:**
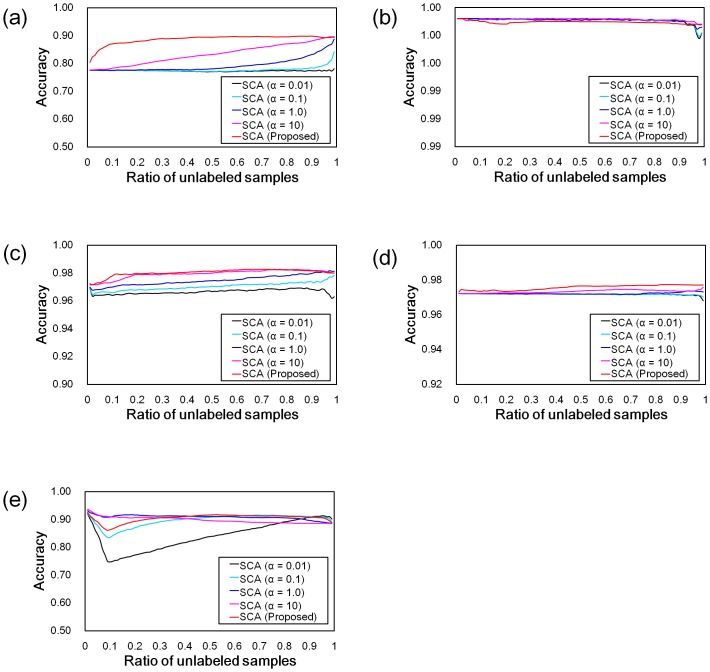
Recognition rates in UCI machine leaning repository. The results for (a) *Satimage*, (b) *Shuttle*, (c) *Optdigits*, (d) *Pendigits*, and (e) *Isolet*. The lines show the classification accuracies in the transformed feature spaces for each method. The colors indicate the transformation methods, as shown in the legend.

We also compared the stability of the features transformed by SCA at each *α*. Fig 1 shows the mean recognition rates in the range of 0.01 ≤ *β* ≤ 0.99, and the experimental setting was same as Table 2. For almost all datasets, the SCA by setting our proposed parameter produced the more stable changes of mean recognition rates than that by other *α*. For *Isolet*, the mean recognition rates produced by SCAs (*α* was set to 0.01, 0.1, and *tr*(Σ) / *m*) unfortunately decreased around *β* = 0.10, when the other SCAs (*α* was set to over 1.0) produced the stable changes. However, the proposed parameter was able to reduce this decreasing in comparison with the parameters which was set to 0.01 and 0.1, and our proposed SCA produced the best results of *Satimage*. From the results, our proposed SCA achieves the relatively stable feature transformation compared with the SCAs by setting to the given *α*.

We can perceive that the results in Table 2 and Fig 1 have a tendency of which the mean recognition rates increase in proportion to *α* and *β*. The tendency suggests that a distinguishability of all samples may be more effective than the within-class coherency in the classification by using the NN classifier, because we can interpret that the distinguishability is proportional to *α* and *β* as shown in Eq (17). From the discussion, SCA and also PCA would produce the better recognition rates than LDA, when we solve the classification problems based on NN.

To confirm the above discussion, SCA was compared to the other feature transformation methods based on the multivariate analysis methods; PCA [12, 13], LPP [20, 21], LDA [15], and SDA [19]. Where PCA and LPP are the unsupervised methods, LDA is the supervised method, and SDA, and also SCA, are the semi-supervised methods. In this experiment, PCA, LPP, and LDA were applied to the datasets to confirm the baselines of recognition rates produced by the unsupervised methods and the supervised method.

The similarity measures used for LPP and SDA were the same as those used in [21] and [17], respectively. The hyper parameters in LPP and SDA were tuned by a grid search with five-fold cross validation (CV) with training dataset. The grid was set to {2^−15^, 2^−14^, 2^−13^, …, 2^15^}. For the unsupervised methods, PCA, LPP, and SCA (*β* = 1), the transformation matrices were estimated by using the training samples without class labels. The transformation matrices by LDA, SDA (*β* = 0), and SCA (*β* = 0) were estimated by using the all labeled training samples. In those cases, we reported the classification accuracies on the given train/test splits. When *β* was set in the range from 0.01 to 0.99, the results of semi-supervised methods, SDA and SCA, were the mean recognition rates and standard deviations as with Table 2.

Table 3 shows the recognition rates for the PCA, LPP, LDA, and shows the highest recognition rates and the standard deviations for each of the semi-supervised methods. For all datasets, the SCA produced the highest recognition rates. Almost all the results of LPP, especially for *Satimage* and *Isolet*, produced the lowest recognition rates in each of the methods. The results of LPP might be cause by the similarity measure which was not suitable for these datasets. The results of SDA had the comparable or better recognition rates than those of LDA, and the graph of SDA for unlabeled samples was based on the similarity measure which was not same as LPP. These results of LPP and SDA suggest the difficulty of the similarity measure selection in the classification tasks. For *Satimage* and *Pendigits*, the ratios of unlabeled samples in SCA were over 0.8, and the PCA produced the higher recognition rates than other methods excluding SCA. On the other hand, for *Shuttle* and *Isolet*, the LDA and SCA (*β* = 0) produced the best recognition rates. From the results, the size of *β* in SCA indicates the effectiveness of distinguishability in the classification based on NN.

**Table 3:** Recognition rates with benchmark dataset.

	*Satimage*	*Shuttle*	*Optdigits*	*Pendigits*	*Isolet*
PCA [*β* = 1]	0.8925	0.9989	0.9977	0.9771	0.8698
LPP [*β* = 1]	0.5500	0.9809	0.9254	0.9731	0.2996
LDA [*β* = 0]	0.8330	**0.9992**	0.9572	0.9523	**0.9384**
SDA	0.8861 (± 0.0021) [*β* = 0.01]	**0.9992** (± 0.0001) [*β* = 0.84]	0.9664 (± 0.0014) [*β* = 0.15]	0.9665 (± 0.0035) [*β* = 0.97]	0.9321 (± 0.0021) [*β* = 0.05]
**SCA**	**0.8985** **(± 0.0008)** **[*β* = 0.90]**	**0.9992** [*β* = 0]	**0.9827** **(± 0.0007)** **[*β* = 0.68]**	**0.9774** **(± 0.0001)** **[*β* = 0.82]**	**0.9384** [*β* = 0]

These results suggest that the proposed SCA is likely to transform the data into discriminating features those are useful for classification tasks.

### Application to tissue image classification

**Fig 2:**
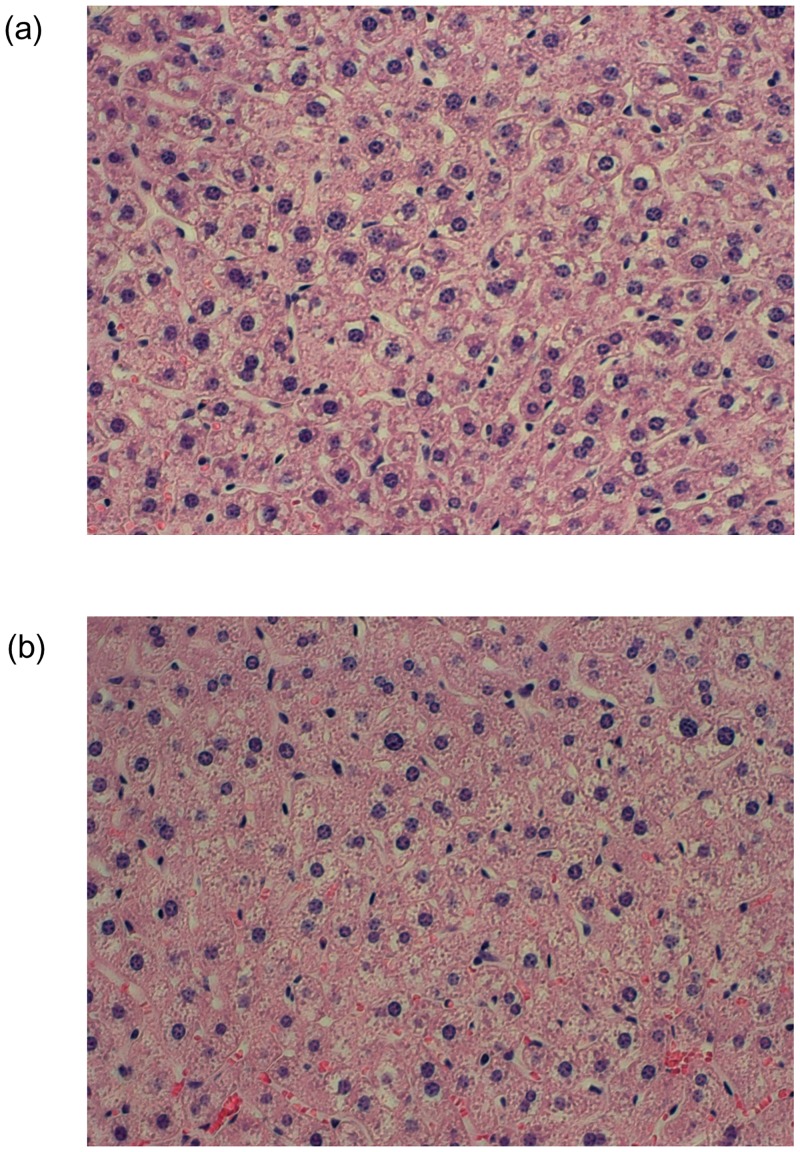
Examples of tissue images. (a) Tissue image for a 24-month-old female mouse on an ad libitum diet. (b) Tissue image for 6-month-old female mouse on a calorie-restricted diet.

We applied our proposed SCA to three tissue image classifications; we used the Image Informatics and Computational Biology Unit (IICBU) 2008 dataset [24], which has been proposed as a benchmark for testing and comparing the performance of analysis methods for biological imaging. This database contains eleven subsets, each representing a different classification problem; the *Liver gender (caloric restriction; CR)*, *Liver gender (ad libitum; AL)*, and *Liver aging* datasets pose particularly difficult problems [5] for tissue image classification. Table 4 presents a summary of these datasets, and examples of tissue images are shown in Fig 2. We applied the various feature transformation methods to these difficult problems.

**Table 4:** Tissue image datasets in IICBU 2008.

	# of samples (images)	# of class	Image format
*Liver aging*	529	4	1,388 × 1,040 RGB TIFF, 12 bit color channel
*Liver gender (CR)*	303	2	1,388 × 1,040 RGB TIFF, 12 bit color channel
*Liver gender (AL)*	265	2	1,388 × 1,040 RGB TIFF, 12 bit color channel

When we directly classify images by using a classifier, it is generally difficult to achieve a favorable classification performance. In order to overcome this problem, various feature extraction methods have been proposed in Computer Vision, and we may improve the classification accuracies by transforming the extracted features into the discriminating features. The gist global descriptor [25], simply called “GIST”, is one of the popular feature extraction methods. The GIST showed better recognition performances than other state-of-the-art methods such as the bag of features (BOF) [26]. The GIST extraction software in [26] is available online [27], and we can readily apply this software to extract the GIST from the color image. In this paper, we extract the GIST from each of the tissue images, and the feature transformation methods were applied to those GISTs.

In the experiments described below, the GIST was directly extracted from each of the color images. The given parameters of GIST were set to the defaults of software [27], resulting a 960-dimensional feature vector. The classification accuracies were evaluated by using the stratified five-fold CV. In each validation set, the extracted GISTs were transformed by using the kernel SCA (KSCA) and the other feature transformation methods; kernel PCA (KPCA), kernel LPP (KLPP), kernel DA (KDA), and kernel SDA (KSDA), those were conducted in the linear space by solving the dual formulation to deal with the ill-posed problem. The settings for the classifier, the similarity measures for KLPP and KSDA, and the method for determining the parameters in each of validation sets were the same as in the previous section.

**Fig 3:**
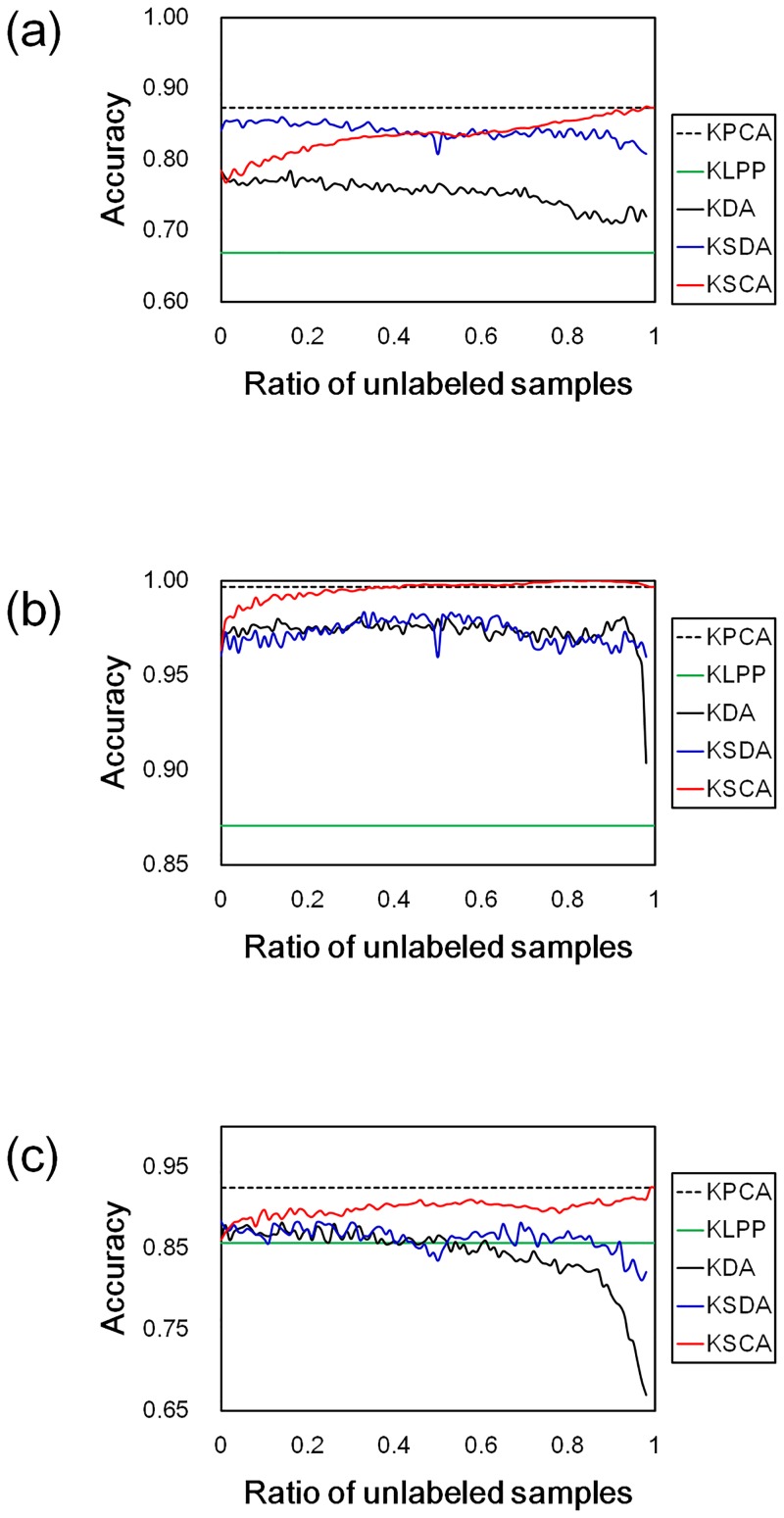
Recognition rates in IICBU 2008 by using NN classifier. The results for (a) *Liver aging*, (b) *Liver gender (CR)*, and (c) *Liver gender (AL)*. The lines show the classification accuracies in the transformed feature spaces for each method. The colors indicate the feature transformation methods, as shown in the legend.

Table 5 shows the mean recognition rates and the standard deviations for the KPCA, KLPP, KDA, and shows the highest mean recognition rates and the standard deviations for each of the semi-supervised methods, in which those were evaluated by using the stratified five-fold CV. Fig 3 shows the mean recognition rates at each *β*. For KDA in Fig 3, the labeled samples from the result of random splits were used for the training at each *β*. KSCA produced the best recognition rates excluding the result for *Liver gender (AL)*. For *Liver gender (AL)*, the recognition rates by KPCA and KSCA show 0.925, and are better results than those by the other feature transformation methods, but the best recognition rate is the result of directly using the GIST. These would be caused from a decreasing of classification performance due to the excessive dimensionality reduction, because the transformed features, especially in the transformation by KDA, were low-dimensional vectors compared with the input features.

**Table 5:** Recognition rates with IICBU 2008 by calculating NN classifier.

	*Liver aging*	*Liver gender (CR)*	*Liver gender (AL)*
GIST	0.873 (± 0.042)	0.925 (± 0.024)	**0.997 (± 0.007)**
GIST + KPCA [*β* = 1]	0.873 (± 0.042)	0.997 (± 0.007)	0.925 (± 0.024)
GIST + KLPP [*β* = 1]	0.669 (± 0.067)	0.871 (± 0.059)	0.857 (± 0.035)
GIST + KDA [*β* = 0]	0.785 (± 0.039)	0.963 (± 0.040)	0.860 (± 0.046)
GIST + KSDA	0.860 (± 0.040) [*β* = 0.14]	0.983 (± 0.018) [*β* = 0.33]	0.883 (± 0.032) [*β* = 0]
GIST + **KSCA**	**0.875 (± 0.036)** [*β* = 0.98]	**1.000 (± 0.000)** [*β* = 0.79]	0.925 (± 0.024) [*β* = 0.99]

In the classification, KDA generally produces higher recognition rates than KPCA. However, in Table 5 and Fig 3, KPCA, and also KSCA, produced the better recognition rates than KDA. These results would be caused by the instance-based classifier. To confirm the effects of classifier on the tissue image classifications, we conducted the experiments only replacing the NN classifier with the nearest mean (NM) classifier which is one of the simple model-based classification methods. The NM classifier can reduces the computation time comparing with the NN classifier.

**Fig 4:**
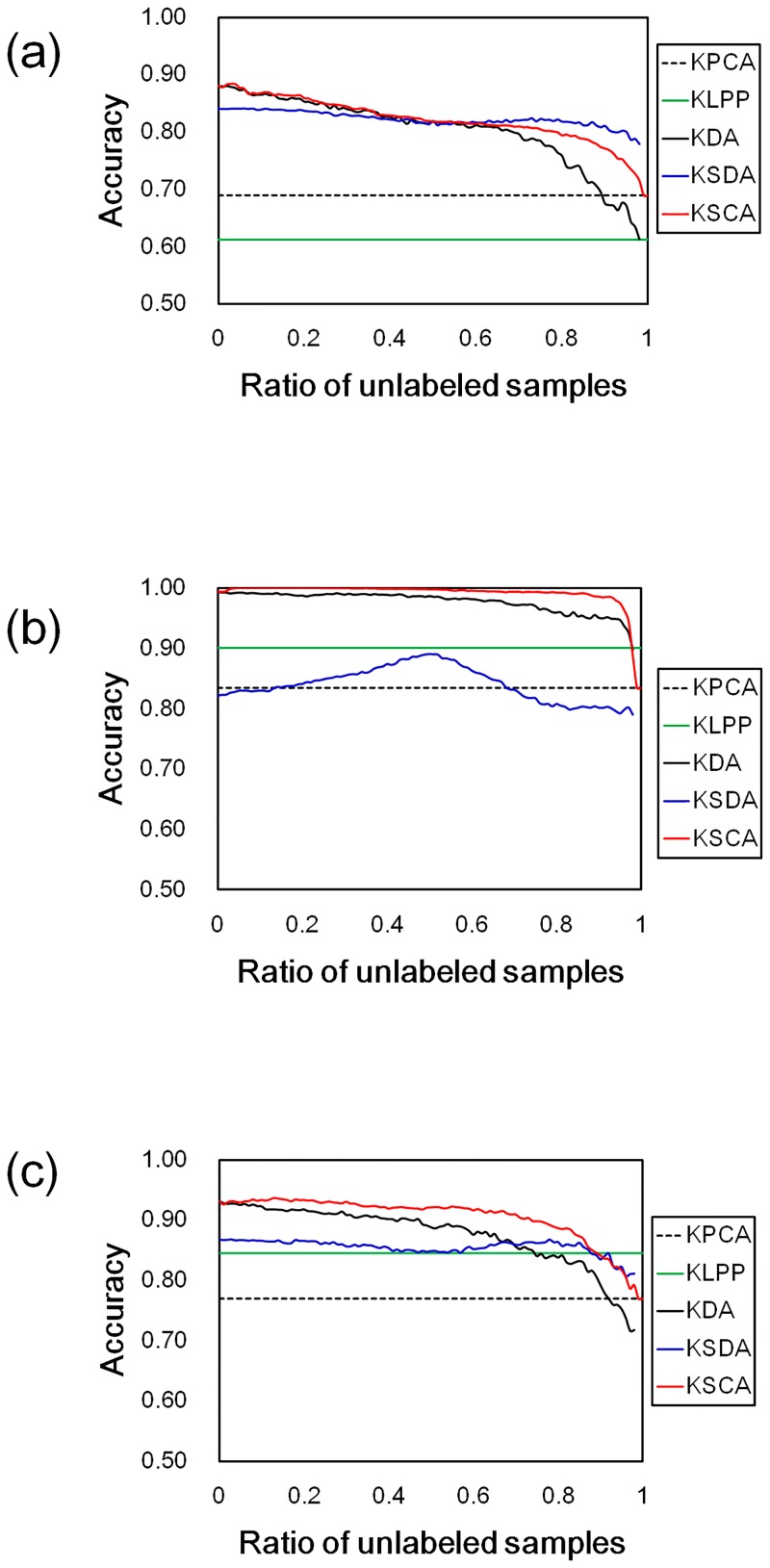
Recognition rates in IICBU 2008 by using NM classifier. The results for (a) *Liver aging*, (b) *Liver gender (CR)*, and (c) *Liver gender (AL)*. The lines show the classification accuracies in the transformed feature spaces for each method. The colors indicate the feature transformation methods, as shown in the legend.

Fig 4 shows the classification accuracies, and the experimental setting in Fig 4 was same as that in Fig 3 except that the test datasets in each of validation sets was classified by using the NM classifier. Table 6 shows the highest mean recognition rates and the standard deviations for each the method in Fig 4.

**Table 6:** Recognition rates with IICBU 2008 by calculating NM classifier.

	*Liver aging*	*Liver gender (CR)*	*Liver gender (AL)*
GIST	0.690 (± 0.069)	0.834 (± 0.032)	0.770 (± 0.032)
GIST + KPCA [*β* = 1]	0.690 (± 0.069)	0.834 (± 0.032)	0.770 (± 0.032)
GIST + KLPP [*β* = 1]	0.613 (± 0.072)	0.901 (± 0.051)	0.845 (± 0.030)
GIST + KDA [*β* = 0]	0.881 (± 0.035)	0.993 (± 0.008)	0.932 (± 0.026)
GIST + KSDA	0.841 (± 0.070) [*β* = 0.06]	0.890 (± 0.035) [*β* = 0.50]	0.868 (± 0.0231) [*β* = 0.03]
GIST + **KSCA**	**0.884 (± 0.040)** [*β* = 0.03]	**1.000 (± 0.000)** [*β* = 0.06]	**0.937 (± 0.016)** [*β* = 0.13]

**Fig 5:**
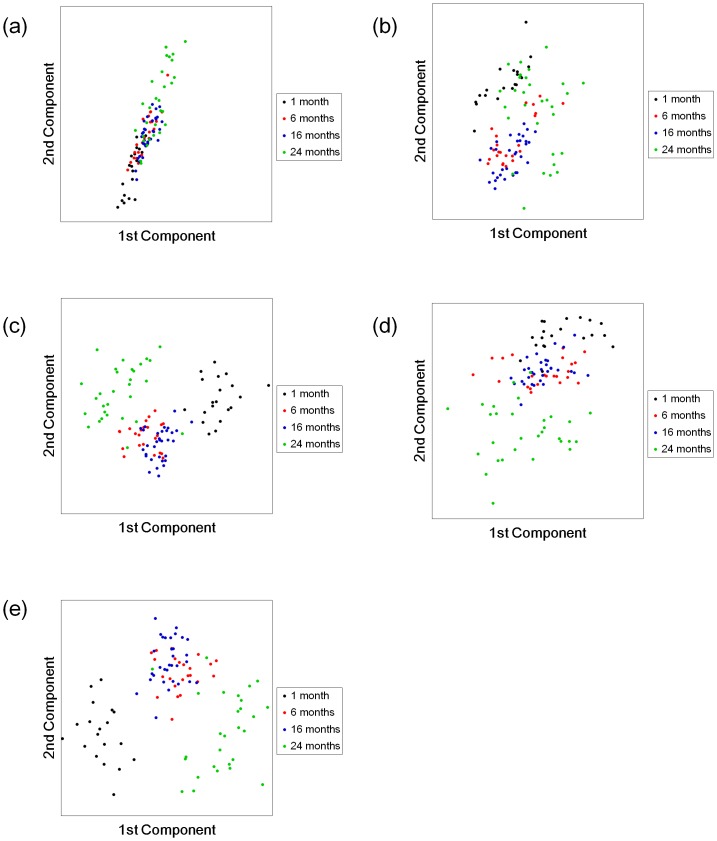
Examples of transformed feature spaces for test samples in the *Liver aging* data. The feature spaces as transformed by (a) KPCA, (b) KLPP, (c) KDA, (d) KSDA (*β* = 0.06), and (e) our proposed KSCA (*β* = 0.03). The *x-* and *y*-axes show the transformed first and second components, respectively, of a validation set in Table 6. The colors indicate the class labels, as shown in the legend.

The KDA and KSCA produced the better recognition rates than the unsupervised methods when *β* was set in the range from 0.0 to about 0.9 as shown in Fig 4, and the results of KDA, KSDA and KSCA had better recognition rates than those of other methods as shown in Table 6. These results are consistent with the sample distributions in the transformed feature spaces as shown in Fig 5. From the results, the model-based classifier shows the favorable classification results when we want to keep the consistency between the recognition rate and the sample distribution.

The results of KSCA had the best recognition rates for all datasets in Table 6, and the results of KSCA in Table 6 had the comparable or better recognition rates than those in Table 5. Moreover, as shown in Figs 3 and 4, KSCA produced the comparable or better classification accuracies than KDA without depending on the ratio of unlabeled samples in training data. From the results, the distinguishability in our proposed method would improve the classification accuracy regardless of the classifiers.

These results suggest that our proposed method with a discriminating feature transformation method results in more accurate tissue image classification than do the various other multivariate analysis methods.

**Fig 6:**
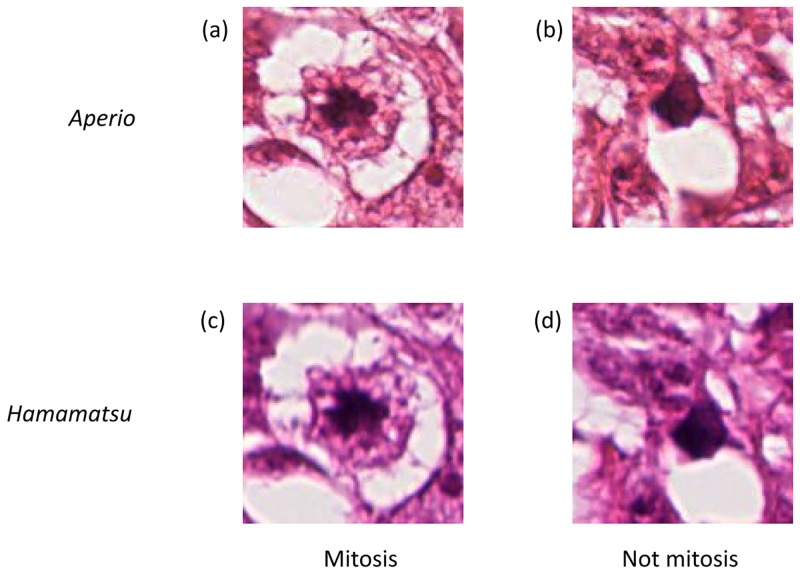
Examples of Mitosis and Not mitosis images. (a) The cropped image for “Mitosis” scanned by *Aperio*. (b) The cropped image for “Not mitosis” scanned by *Aperio*. (c) The cropped image for “Mitosis” scanned by *Hamamatsu*. (d) The cropped image for “Not mitosis” scanned by *Hamamatsu*.

We also applied our proposed SCA to other biological image dataset, MITOS-ATYPIA-14, which was released in the MITOS & ATYPIA Contest [28]. This dataset contains the breast cancer biopsy slide images which were scanned by two slide scanners: Aperio Scanscope XT (*Aperio*) and Hamamatsu Nanozoomer 2.0-HT (*Hamamatsu*). The annotation for the images represents the two-class classification problem; “Mitosis” or “Not mitosis” as shown in Fig 6. In this paper, we used MITOS-ATYPIA-14 to evaluate the classification accuracies from the precise annotation, though the dataset had been released to evaluate detection accuracies in the MITOS & ATYPIA Contest. Table 7 shows the summary of this dataset for classification.

**Table 7:** Tissue image datasets in MITOS-ATYPIA-14.

Scanner	# of scanned images	Scanned image size	# of cropped images
*Aperio*	1,200	1,539 × 1,376 pixels	749 (Mitosis) 2,884 (Not mitosis)
*Hamamatsu*	1,200	1,663 × 1,485 pixels	753 (Mitosis) 2,891 (Not mitosis)

In the experiments described below, the setting of evaluation for classification accuracies, the similarity measures for LPP and SDA, and the method for determining the parameters in each of validation sets were the same as in the above experiments, while applying the NN classifier. The classification accuracies were measured on the stratified five-fold CV. The sample images (image size: 128 × 128 pixels) were cropped from the scanned images as shown in Fig 6, and the 960-dimensional GIST features were extracted from each of the cropped images. The parameter setting of GIST was the same as in the above experiments.

Table 8 shows the highest mean recognition rates and the standard deviations for each of the semi-supervised methods, and shows the mean recognition rates and the standard deviations for the other methods. In each of the scanners, LPP (for *Aperio*) and SDA (for *Hamamatsu*) produced the best recognition rates than other feature transformation methods, respectively. However, the recognition performances by LPP for *Hamamatsu* and SDA for *Aperio* decrease by about 0.03 which is larger than those by the methods. These would be caused by the similarity measure, and these results imply the difficulty of similarity measure selection as is the case with the results in Table 3. In contrast, SCA produced the better performances than PCA and LDA with slightly changing recognition rates for each scanner. These results suggest that SCA can produce the favorable feature transformation performance in disregard of the scanner types.

**Table 8:** Recognition rates with MITOS-ATYPIA-14 by using GIST features.

	*Aperio*	*Hamamatsu*
GIST	0.675 (± 0.017)	0.659 (± 0.010)
GIST + PCA [*β* = 1]	0.676 (± 0.015)	0.658 (± 0.009)
GIST + LPP [*β* = 1]	**0.794 (± 0.001)**	0.763 (± 0.016)
GIST + LDA [*β* = 0]	0.747 (± 0.026)	0.730 (± 0.019)
GIST + SDA	0.760 (± 0.012) [*β* = 0.02]	**0.791 (± 0.002)** [*β* = 0.99]
GIST + SCA	0.754 (± 0.008) [*β* = 0.24]	0.749 (± 0.011) [*β* = 0.06]

From the above experiments, we presented that SCA, and also the other feature transformation methods, can improve the classification accuracies by combining with GIST. SCA would improve the classification accuracy without depending on types of feature extraction methods and slide scanners. To confirm this claim, we further conducted the following experiments changing the feature extractor from GIST to CNN.

Table 9 shows the highest mean recognition rates and the standard deviations for SCA, and shows the mean recognition rates and the standard deviations for direct classification by using CNN features, in which those were evaluated by using the stratified five-fold CV with the NN classifier. We used the Alex CNN model [29] and employed as feature extractors FC6 layer of the CNN pre-trained on the ImageNet dataset [9]. The sample images (image size: 256 × 256 pixels) were cropped from the scanned images, and the 4096-dimensional CNN features were extracted from each of the cropped images. The dimension of CNN features in MITOS-ATYPIA-14 is larger than the number of samples. To deal with this ill-posed problem, we applied KSCA as shown in the method section. The direct classifications by CNN features show the better recognition rates than those by GIST features, and SCA further improved the classification accuracies as shown in Table 9. These results suggest that SCA can improve the classification accuracies without depending on the types of feature extraction methods and slide scanners.

**Table 9:** Recognition rates with MITOS-ATYPIA-14 by using CNN features.

	*Aperio*	*Hamamatsu*
CNN	0.718 (± 0.012)	0.723 (± 0.008)
CNN + KSCA	0.776 (± 0.015) [*β* = 0.03]	0.775 (± 0.012) [*β* = 0.02]

## Conclusion

We proposed a semi-supervised feature transformation method, and we applied it to the classification of tissue images. Our proposed method, semi-supervised component analysis (SCA), was inspired by PCA and LDA in the graph-embedding framework. SCA produced better classification performances than did other feature transformation methods for the benchmark datasets from the UCI machine learning repository. Furthermore, the kernel extended SCA contributed better classifications of tissue images in the IICBU 2008 and the MITOS-ATYPIA-14.
